# Identification of depression subtypes and relevant brain regions using a data-driven approach

**DOI:** 10.1038/s41598-018-32521-z

**Published:** 2018-09-20

**Authors:** Tomoki Tokuda, Junichiro Yoshimoto, Yu Shimizu, Go Okada, Masahiro Takamura, Yasumasa Okamoto, Shigeto Yamawaki, Kenji Doya

**Affiliations:** 1 Okinawa Institute of Science and Technology Graduate University, 1919-1 Tancha, Okinawa, 904-0495 Japan; 2 Department of Psychiatry and Neurosciences, Hiroshima University, 1-2-3 Kasumi, Minami-ku, Hiroshima, 734-8553 Japan; 3 Graduate School of Information Science, Nara Institute of Science and Technology, 8916-5 Takayama, Ikoma, Nara, 630-0192 Japan

## Abstract

It is well known that depressive disorder is heterogeneous, yet little is known about its neurophysiological subtypes. In the present study, we identified neurophysiological subtypes of depression related to specific neural substrates. We performed cluster analysis for 134 subjects (67 depressive subjects and 67 controls) using a high-dimensional dataset consisting of resting state functional connectivity measured by functional MRI, clinical questionnaire scores, and various biomarkers. Applying a newly developed, multiple co-clustering method to this dataset, we identified three subtypes of depression that are characterized by functional connectivity between the right Angular Gyrus (AG) and other brain areas in default mode networks, and Child Abuse Trauma Scale (CATS) scores. These subtypes are also related to Selective Serotonin-Reuptake Inhibitor (SSRI) treatment outcomes, which implies that we may be able to predict effectiveness of treatment based on AG-related functional connectivity and CATS.

## Introduction

Major depressive disorder (MDD) is a common, but serious disorder, characterized by severe symptoms that affect how one feels, thinks, and manages daily activities^1^. It is estimated that 17% of all people experience MDD at one point or another during their lifetimes^2^. Despite its prevalence, however, clinical diagnosis is rather limited, and is based on highly subjective criteria. Current diagnostic practice relies on clinical questionnaires in which patients reply to questions on a wide range of depression-related items listed in the Diagnostic and Statistical Manual of Mental Disease (DSM)^3^. Although DSM provides a coherent framework for diagnosis, it remains subjective due to its dependence on patient responses. In addition to subjectiveness, diagnosis involves time-consuming interviews, which require both professional medical expertise and close cooperation of a patient^4^.

To improve diagnostic objectivity, much attention has recently been paid to potential biomarkers for depression. In particular, a number of studies have focused on neural activities in the brain, measured as functional magnetic resonance imaging (fMRI) signals^5–7^. Typically, relevant brain areas for depression are identified in a supervised manner, i.e., using labels of control/depression that are subsequently evaluated for diagnosis of depression in a test dataset. In this framework, the binary label of control/depression plays a key role in identification of these brain areas.

A more challenging problem in MDD is the identification of its subtypes. It is well known that MDD is heterogenous in such characteristics as clinical presentation, progression, treatment response, genetics, and neurobiology^8^. This heterogeneity hampers progress in identifying the cause of MDD and its effective treatment^9^. To overcome this problem, several studies have been conducted to identify subtypes of MDD in a data-driven manner, relying on clinical questionnaires^10,11^. However, the results of these studies either conflict or they simply identify clusters related to depression severity, which does not provide conclusive evidence for subtypes of depressive symptoms^8^. Furthermore, these studies are based on clinical questionnaires without taking into account biological substrates.

Recently, resting state fMRI, in which a subject does not explicitly perform any task, has increasingly gained attention for MDD prediction^12,13^. Further, several studies using resting state fMRI have shed light on MDD subtypes in terms of treatment resistance^14^. Treatment-resistant depression (TRD) is defined as depression in which clinically meaningful improvement was not observed following the use of two different antidepressants^15^. These studies approached the question of MDD subtypes in a supervised manner (systematic review paper)^16^. Given binary labels of MDD status (TRD or non-TRD), they identified relevant brain areas in which large differences in resting state fMRI images are observed between TRD and non-TRD. It is reported that functional connectivity (FC) increased in the subgenus cingulate and the thalamus^17^, while it decreased in the cerebellum, the precuneus and the inferior parietal lobule for TRD patients^18^. However, some of these results may not be consistent: FC in the right angular gyrus is higher for TRD than non-TRD^19^, while the opposite is reported^18^.

Methodologically, it is more useful to identify MDD subtypes in an unsupervised manner (cluster analysis), because the unsupervised approach can reveal underlying heterogenous subtypes without prior knowledge of subtypes, leading to a better understanding of MDD. However, due to lack of an appropriate statistical method, current research on clustering of subjects based on fMRI data remains limited to the case of binary clustering (MDD, healthy) focused on specific brain areas^20^. It is challenging to directly apply cluster analysis to high-dimensional data, such as whole volume neuroimaging data, because of features possibly irrelevant to a clustering solution that may distort clustering results^21^. To alleviate such a problem, Principal Components Analysis (PCA) is often used for dimensional reduction^22^. Likewise, Canonical Correlation Analysis (CCA) has recently been used^23^. In CCA, a weighted linear combination of features (functional connectivity) is identified to maximize a correlation with another linear combination of clinical indicators that capture phenomenological aspects of depression. In both PCA- and CCA-based approaches, resulting linear combinations of features are subsequently used for further cluster analysis of subjects. However, these approaches may fail to capture underlying cluster structures in high-dimensional data. In such datasets, multiple clustering structures often exist, i.e., different subsets of features are relevant for different clusters. For instance, in a given sample of subjects, we may find a cluster structure of subjects depending on age (together with other features), and this cluster structure might be related to the status of Alzheimer’s disease because this disease is strongly related to aging. Likewise, in the same sample, we may find another cluster structure of subjects depending on sex, which might be related to the status of Thyroid disease because women suffer from this disease more often than men. Nonetheless, PCA or CCA does not identify such multiple view structures of clusters^24^. In the CCA approach^23^, hierarchical cluster analysis is carried out using the first and second principal components, yielding a single cluster solution. Hence, only a dominant cluster structure is captured, while non-dominant cluster structures may be overlooked. However, useful subtypes of depression may be related to a specific subset of brain areas that are not captured by PCA or CCA. Importantly, appropriate feature selection not only captures relevant features, but also eliminates irrelevant features (e.g., sex) that are not related to depression. This enhances quality of clustering and also facilitates interpretations of clustering results in terms of selected features. Nonetheless, it is still a challenge to identify correspondence between subtype solutions and relevant brain areas.

In the present paper, we address this question by directly modeling cluster structures in high-dimensional data. The key idea is to explicitly model multiple cluster solutions that optimally partition features. This has the effect of analyzing only features relevant to a particular cluster solution, while irrelevant features are used for other cluster solutions. We use a recently proposed clustering method for high-dimensional data that yields clustering solutions for optimally selected features^25^. We apply this method to a combination of several dataset modalities, such as FC data in resting state fMRI, clinical questionnaires, and gene expression data.

In this study, to allow for flexible cluster structures with possible overlaps of patients and controls, we include both types of subjects for clustering. The inclusion of both types of subjects enriches interpretations of subject clusters. Further, from the statistical point of view, it is advantageous to ensure larger sample size. As regards patients, to minimize the effect of antidepressants, we focus on patients who were either untreated or were treated with a single drug at insufficient dose and duration. Due to the variety of data modalities, the resulting concatenated dataset includes numerical, categorical and integer features, with or without missing entries. Furthermore, the dataset is high-dimensional with the number of dimensions being larger than 2000. From results of cluster analysis, possible subtypes may be related to functional connectivity, child abuse trauma, and initial depression severity. Using these subtypes, the combination of angular-gyrus-related functional connectivity and child abuse trauma is useful for predictions on remission of depression by Selective Serotonin Reuptake Inhibitor (SSRI) treatment.

## Data

Our data comprise several modalities, such as FC data in resting state fMRI, clinical questionnaires, and biological data. For simultaneous analysis of all datasets, we concatenated them into a single data matrix (subjects are common to the sub-datasets). Prior to collecting these data, this study was approved by the Research Ethics Committee at the Okinawa Institute of Science and Technology, as well as the Research Ethics Committee of Hiroshima University (permission nr.172). All research was performed in accordance with the relevant guidelines and regulations. Informed written consent was obtained from all participants in the study (approved by both institutions). In the following subsections, more detailed descriptions of our datasets are provided.

### Subjects

Sixty-seven patients aged 26–63 (average 40.43 ± 9.75 (s.d.), 34 males and 33 females) with MDD were recruited by the Psychiatry Department of Hiroshima University and collaborating medical institutions, based on the Mini International Neuropsychiatric interview^26^, which enables medical doctors to identify psychiatric disorders according to the Diagnostic and Statistical Manual of Mental Disorders, Fourth Edition^3^. Their scores of Beck Depression Inventory (BDI)^27^ ranged from 16 to 53 with average 32.10 ± 9.05 (s.d.) while those of the Japanese version of the national adult reading test (JART)^28^, which was used to estimate intelligence quotient (IQ), ranged from 87.6 to 125.7 (average 110.0 ± 9.6 (s.d.)). These demographic and clinical characteristics are summarized in Table 1. These patients were either untreated or treated with a single antidepressant at insufficient dose and duration. Additional inclusion criteria were age between 25 and 80 and written informed consent. Exclusion criteria entailed current or past manic episodes; psychotic episodes; alcohol dependence or/and abuse; substance dependence or/and abuse; antisocial personality disorder. As a control group, 67 subjects aged 20–66 (average 36.58 ± 12.03 (s.d.), 30 males and 37 females) with no history of mental or neurological disease were recruited using advertisements in local newspapers. All control subjects underwent the same self-assessment and examination that was administered to the MDD group. Control subjects also undertook the Mini International Neuropsychiatric interview by experienced psychiatrists and psychologists, to ensure that they did not have any previous or current psychiatric disorder according to DSM-IV criteria. For control subjects, BDI scores ranged from 0 to 42 (average 7.35 ± 7.18 (s.d.)) while JART scores ranged from 84.7 to 126.5 (average 111.8 ± 8.5 (s.d.)). Here, too, all subjects gave written informed consent. To compare demographic and clinical characteristics between depressive and control subjects (Table 1), the results of p-value suggest that for sex and IQ the difference between two groups is not significant (p-values are 0.48 and 0.21, respectively), whereas for age and BDI the difference is significant (p-values are 0.014 and 2.2 × 10^−21^, respectively). Both depressive and control subjects underwent MRI acquisition with a GE scanner. Note that fMRI image data for depressive subjects were obtained within two weeks after the onset of treatment with SSRI.

**Table 1:** Demographic and clinical characteristics of subjects.

	MDD	Control	P-value for the difference
Number of subjects	67	67	NA
Sex (male/female)	34/33	30/37	0.48
Age (mean, s.d.)	40.43 (9.75)	36.58 (12.03)	0.014*
BDI (mean, s.d.)	32.10 (9.05)	7.35 (7.18)	2.2 × 10^−21^***
IQ (mean, s.d.)	110.0 (9.6)	111.8 (8.5)	0.21

P-value is evaluated for the difference between MDD and Heathy Control for each characteristic. For sex, we performed the *χ*-square test, whereas for Age, BDI scores and IQ, we performed the two-sided *U*-test (Wilcoxon-Mann-Whiteny test). Asterisks denote level of significance of p-values: ***p < 0.001; **p < 0.01; *p < 0.05

### Functional MRI data

fMRI measurements were performed at Hiroshima University on a 3T GE Signa HDx scanner with GE-EPI (TR = 2 s, 150 volumes = 5 min scan, TE = 27 ms, FA = 90°, matrix size 64 × 64 × 32, voxel size 4 × 4 × 4 mm, no gap, interleaved). We discarded initial five volumes of the scanned data. Structural T1 images were acquired after the fMRI experiments for correction of head position changes in the subsequent analysis (IRP FSPGR, TR = 6.824 ms, TE = 1.9 ms, FA = 20°, FOV = 256 mm, matrix size 256 × 256 × 180, voxel size 1 × 1 × 1 mm). For scanning of fMRI, the following common settings and instructions were used. In the scan room with dimmed lights, subjects were asked not to think of nor to sleep, but keep looking at a cross mark in the center of the monitor screen. For the pre-processing of the fMRI data, images were realigned, normalized and smoothed (FWHM = 8 mm) using SPM8 (Statistical Parametric Mapping 8)^29^. They were band-pass filtered (0.009–0.1 Hz) and de-trended.

Using these fMRI measurements, functional connectivity between regions of interest (ROIs) were evaluated. In the present study, we used a common definition of functional connectivity: correlation coefficient of average time series of BOLD fMRI signals between ROIs^30^. For ROIs, we used recently suggested ROIs based on intrinsic connectivity networks^31^, which presumably reflect network structures in the brain. These template ROIs were adopted to our data as follows. First, the ROI template was resampled and the center shifted so that the matrix size matched the size of the acquired data and the resting state fMRI image centers coincided (both ROI template as well as resting state fMRI images are normalized to the standard brain). Second, these voxel time series indicated as belonging to the same area by the template were averaged and the correlation coefficient between these average time series was evaluated.

These ROIs consist of 90 brain regions across 14 intrinsic connectivity networks, from a data-driven approach over several subjects by means of Independent Component Analysis. These intrinsic connectivity networks are as follows (digits in parentheses denote the number of ROIs): Anterior Salience (7), Auditory (3), Basal Ganglia (5), Dorsal Default Mode (9), Language (7), Left Executive Control (6), Precuneus (4), Posterior Salience (12), Right Executive Control (6), Ventral Default Mode (10), Visuospatial (11), Primary Visual (2), Higher Visual (2), and Sensorimotor (6). Specifications of ROIs in the present study were based on publicly available nifti files^32^. Note that in the present paper the numbering that follows these network names exactly match those in these nifti files. In this framework, we selected 78 ROIs, excluding cerebellum-related ROIs, because reliable images were not available for these regions. This resulted in 2701 features (78 × 77/2) for functional connectivity in the present study.

### Clinical questionnaire data

Clinical questionnaire data comprise several types of scores that measure depression severity (e.g., Beck Depression Inventory), personality (e.g., NEO personality Inventory), and life experiences (e.g., Child Abuse Trauma Scale). In particular, Beck Depression Inventory (BDI) and Hamilton Rating Scale of Depression (HRSD) are important indicators for measuring the severity of depression focusing on its several symptoms^8^. Hence, we also include question items about these indicators in our dataset (Supplementary Table S2 for the whole list of features).

The same clinical questionnaires were administered to both depressive and control subjects. Importantly, for depressive subjects, these were administered before the onset of SSRI treatment. Moreover, some indices, such as BDI and HRSD, were again administered six weeks and six months after the treatment (these features are denoted with endings of 6*w* and 6*m* in Supplementary Table S2). We expected that inclusion of repeated measurements in these scores would provide useful information on treatment effect.

### Biological data

Beside functional connectivity factors, we included several biological elements: sex, age, Brain Derived Neurotrophic Factor (BDNF), Cortisol, Single Nucleotide Polymorphisms (SNPs), and DNA Methylation. Specifically, SNPs are for genes relevant to BDNF and serotonin receptors. DNA Methylation is for CpG sites of BDNF receptor genes *trkb* and for serotonin receptor gene *htr2c*. Regarding BDNF and cortisol, measurements after six weeks are also included.

### Pre-processing of data

For functional connectivity data, we standardized each feature using mean and standard deviation of the control group, with the effect that we set the baseline to the average values of control subjects. For the remainder of numerical features, we standardized each feature using its mean and standard deviation, based on all subjects (ignoring missing entries). In contrast, for categorical and integer type of features, we did not carry out pre-processing. Note that we left missing entries as they were, because our clustering method can handle them without explicit substitutions.

## Clustering Method

**Figure 1:**
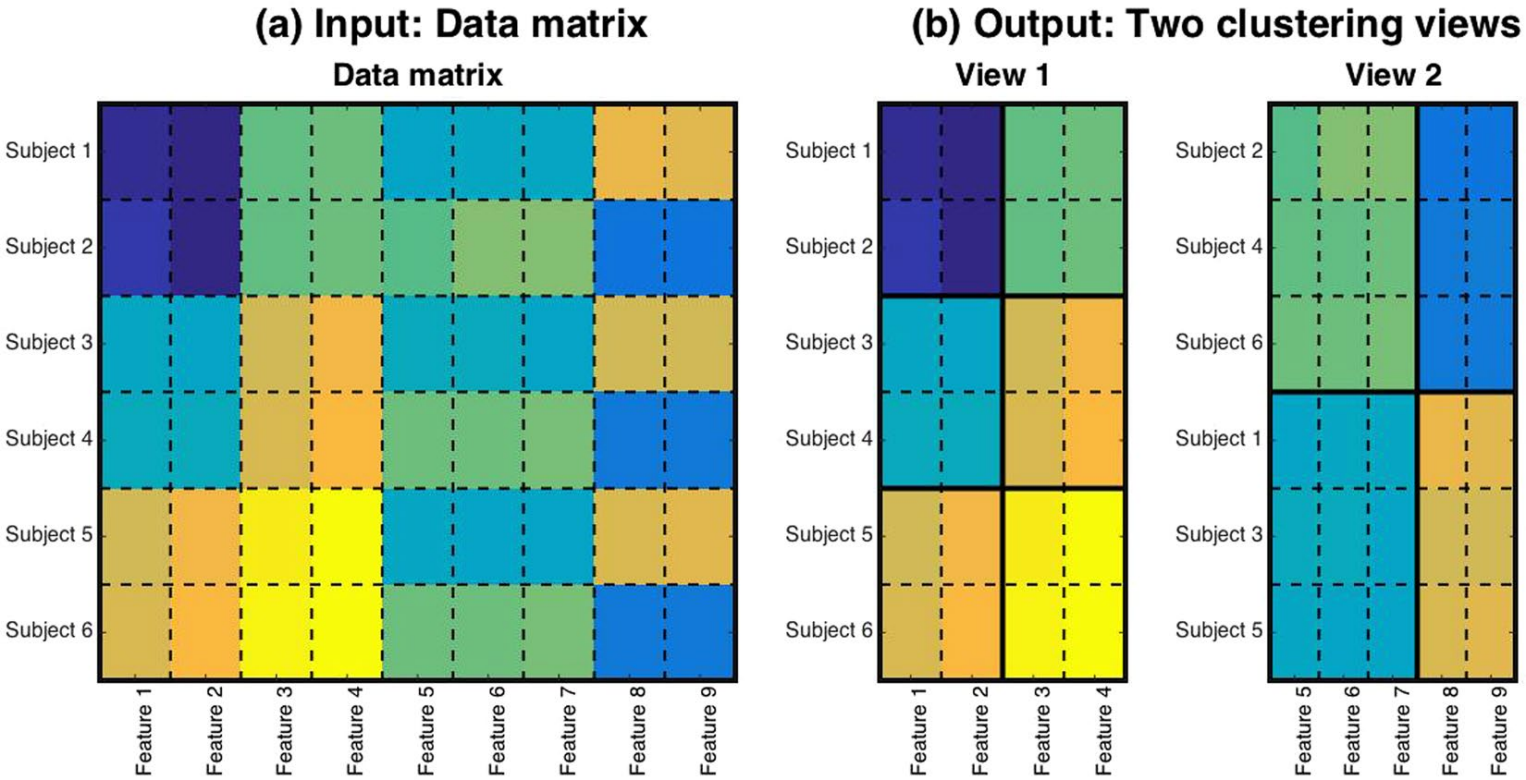
Illustration of the multiple co-clustering method for Input and Output. Panel (a) for an input data matrix and panel (b) for an outcome of the method, which partitions the data matrix into two co-clustering views. The x-axis denotes the feature index while the y-axis denotes the subject index. In panel (b), subjects and features are sorted by their corresponding cluster memberships. Bold lines distinguish between different subject clusters or different feature clusters, while dashed lines distinguish between different subjects or different features. Note that subjects are common across different views, while a particular feature shows up in a view just once because of (exclusive) partition of features among different views.

To conduct cluster analysis, we used a novel method for multiple co-clustering, which we recently developed for purposes of the present study (Supplementary Multiple co-clustering method)^25^. The MATLAB source code for this method is publicly available^33^. Here, the terminology of co-clustering denotes clustering of both subjects and features. The basic assumption of this method is that different subject cluster solutions may be associated with a specific set of features. Here, we refer to a way of looking at subject clusters (depending on relevant features) as a ‘view’. Since the number of combinations of features is exponential in nature, many views are theoretically possible. However, we aim to identify only those that are probabilistically optimal under the non-overlapping constraint of feature selection (i.e., features are partitioned into these views). Our clustering method is based on nonparametric Bayesian statistics^34^, which yield multiple views of co-clustering solutions in which each co-clustering solution provides both feature- and subject-clusters. Specifically, by this method an input is a data matrix while an output is view structures (Fig. 1a,b). The key idea of the method is to optimally partition a data matrix into three folds. First, it partitions features into several views, which works as feature selection for different subject cluster solutions. Second, it further partitions features within a view, which bundles similar features. This feature partition in a view is helpful for avoiding the problem of over-fitting in the case of high-dimensional data, and also for interpreting relevant features. Third, it partitions subjects in each view, which yields several subject cluster solutions. These three phases of partitioning are carried out simultaneously, which yields optimal cluster solutions. In short, this method yields optimal subject cluster solutions by simultaneously selecting corresponding sets of relevant features. Nonetheless, cluster solutions yielded by this method may differ, depending on the initial configuration of the phase partitioning. Hence, in order to prevent local optima, as a conventional practice, we apply this method a number of times with different initial configurations, and choose the best clustering solution in terms of data fitting.

For application to a real dataset, this clustering method has several advantages. First, the number of views, the number of feature clusters, and the number of subject clusters are all automatically inferred based on Dirichlet process^35^. Hence, it is computationally quite efficient. Second, this method can deal with different types of features by pre-specifying possible types of the underlying distribution in a cluster block, i.e., Gaussian, categorical and Poisson. We refer to these types as numerical, categorical, and integer hereafter. Third, it handles missing values in a natural manner in the framework of Bayesian inferences. These characteristics for clustering are specific to this method, which is perfectly suited for analysis of our dataset. Note that a non-Gaussian distribution in numerical features such as a log-normal distribution can be fitted by this method in form of several clusters^36^. This flexibility in fitting a non-Gaussian distribution is advantageous because it wides applicability of the method. Nonetheless, it requires some care for interpreting cluster results whether obtained clustering results represent the underlying cluster structure.

To apply this clustering method to our data, it is required to pre-specify feature types. For our data, functional connectivity and various psychiatric scores are considered as numerical features (i.e., Gaussian distribution is assumed in each cluster block). In contrast, sex and SNPs are considered categorical. With regard to items BDI and HRSD, these are ordinal categorical data. If we considered these as numerical, we would very poorly fit a Gaussian distribution to the data. Hence, we simply considered these items as categorical. This does not jeopardize interpretation of the solutions. Further, the number of depression episodes and repetitions are integers (see Supplementary Table S2 for more details). As a result, our data consists of 2832 numerical features, 114 categorical features, and 2 integer features.

Regarding prior distributions, we consider non-informative priors, setting hyperparameters as follows (we use the notation in the cited paper)^25^: For stick-breaking process, *α*_1_ = *α*_2_ = *β* = 1 in Beta(·|1, *α*_1_), Beta(·|1, *α*_2_), and Beta(·|1, *β*), respectively; for variances of numerical features, *γ*_0_ = 1/100 and ${\sigma }_{0}^{2}=\mathrm{1/100}$ in $\,{\rm{Ga}}(\,\cdot \,|{\gamma }_{0}\mathrm{/2,}{\gamma }_{0}{\sigma }_{0}^{2}\mathrm{/2)}$; for means of numerical features, *μ*_0_ = 0 and *λ*_0_ = 1/100 in ${\rm{Gauss}}(\,\cdot \,|{\mu }_{0},({\lambda }_{0}{s}_{v,g,k}{)}^{-1})$; for categorical features, *ρ*_0_ = 1 for ${\rm{Dirichlet}}(\,\cdot \,|{\rho }_{0})$; for integer features, *α*_0_ = *β*_0_ = 1 in Ga(·|α_0_, *β*_0_). With this setting of priors, we performed the method for 1000 initial configurations of clustering, and selected the model that maximized likelihood of clustering solutions. A single run for one initial configuration took about 0.16 hrs, which amounted to 1000 × 0.16 = 160 hrs to complete all computations. We also examined sensitivity of the clustering results to the setting of hyperparameters, which suggested that the results are not sensitive to a small perturbation of our setting of hyperparameters (Supplementary Sensitivity Analysis).

## Results

**Figure 2:**
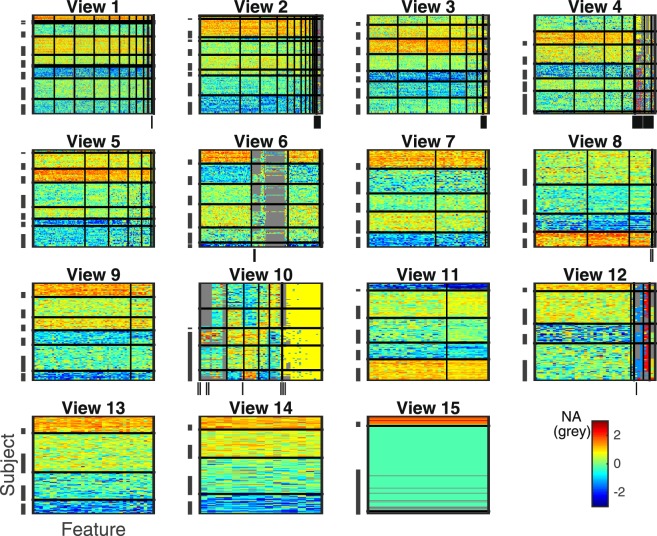
Visualization of all views (view 1–15). The horizontal axis denotes features while the vertical axis denotes subjects. Depressive subjects are indicated by a hyphen while features directly related to diagnose of depression, namely, BDI and HRSD are indicated by a vertical bar. Note that when adjacent subjects or features are indicated by hyphens, these hyphens appear to be merged in a panel. Subject clusters are sorted in ascending order of proportions of depressive subjects from top to bottom. Further, in each subject cluster, control subjects are first displayed. Feature clusters are sorted in order of numerical, categorical and integer types from left to right (separated by thick bold lines). In each type of features, feature clusters are further sorted in descending order of cluster size. Within this sortation, the order of subjects or features is arbitrary. Grey color denotes a missing entry.

**Figure 3:**
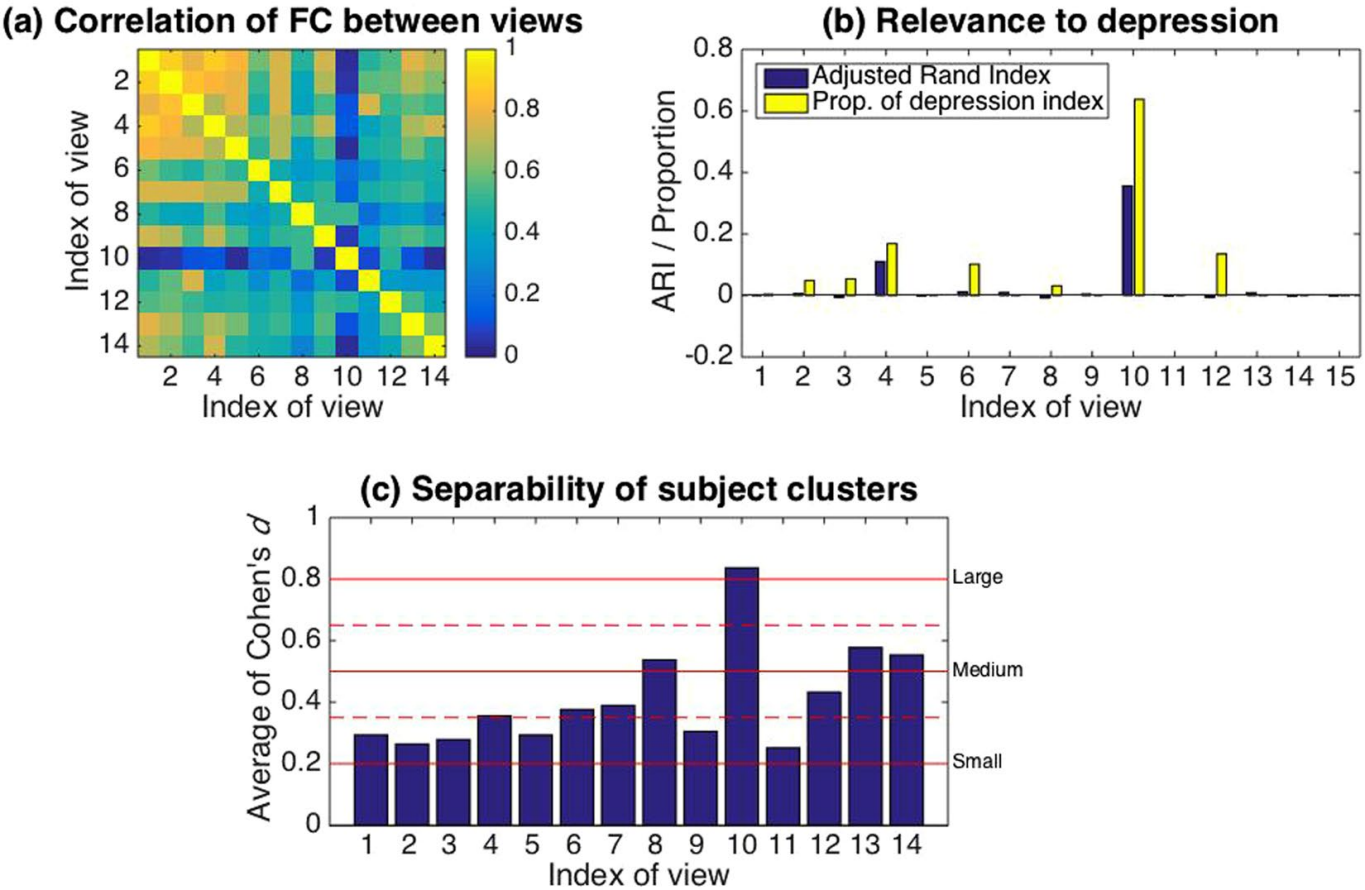
Analysis of feature/subject clusters. Panel (a) Pearson’s correlation coefficients between first principal components of FC features in views. Panel (b): Agreement between the subject cluster and the label of control/depression in blue. The agreement is measured in terms of adjusted Rand Index^52^: zero for no agreement (chance level); one for complete agreement. Also, the proportion of depression-related features (i.e., clinical questionnaire) for selected features is displayed in yellow. Panel (c): Average Cohen’s *d* for FC feature clusters in each view. For each feature cluster, we sorted estimated means in Gaussian component, and evaluated Cohen’s *d*, i.e., $d=({\mu }_{2}-{\mu }_{1})/\sqrt{({\sigma }_{2}^{2}+{\sigma }_{1}^{2}\mathrm{)/2}}$, between neighboring means *μ*_1_ and *μ*_2_ (*μ*_1_ < *μ*_2_). Finally, we took the average of all Cohen’s *d* within a view. In this analysis, we conditioned feature clusters that had more than three FC features, while subject clusters that had more than three depressive subjects. Red bold lines denote levels of separability^37^ while dashed lines denote boundaries between these levels. Note that for view 15, no FC feature is included; hence, we omitted this view from the analysis in Panels (a) and (c).

The multiple co-clustering method yielded 15 views in which there was large variation in the number of features, and the number of subject and feature clusters (Supplementary Table S3; views are sorted in descending order of the number of features). The number of subject clusters ranged from 3 to 9, while the number of feature clusters ranged from 1 to 11 for numerical features, from 1 to 3 for categorical features, and 1 for integer features in case that these types of features may be included in a view. Visual inspection of these views (Fig. 2) shows that the distributions in numerical feature clusters are similar for each subject cluster within a view, except for view 10 (several different features clusters are clearly visible). Further, view memberships of features are displayed in Supplementary Fig. S1 for FC features and in Table 2 for non-FC features. With respect to relationships between views, we focused on the first principal component of numerical features for each view. The first principal component is a weighted linear combination of features that explains most of the variability of features, representing the underlying trend of the data. To compare underlying trends of views, we evaluated Pearson’s correlation coefficient between the first principal components of relevant FC features in each view (Fig. 3a). First principal components are highly correlated between different views, except for view 10, in which the first principal component seems to be independent of those in most of the remaining views (correlated only with views 6, 7, 8 and 12 at significant level 0.05). Note that here we relied on PCA rather than CCA, because CCA focuses on maximum correlation with specific combinations of features while our interest is to evaluate correlations of underlying trends.

**Table 2:** Members of non-FC features in a view.

View ID	Feature types		
	Numerical	Categorical	Integer
View 1	BIS_6w, Cortisol_6w	HRSD_t1_(7, 10, 11), HRSD_t2_(7)	None
View 2	BDNF_6w	BDI_t2_(1–4, 7, 10, 12–16, 18–20),	None
		HRSD_t2_(5, 10, 11, 13)	
View 3	PANAS_N_6w	Remission, BDI_t2(9),	None
		HRSD_t1_(9, 19–21),	
		HRSD_t2_(4, 6, 8, 12, 16–18, 21), **SNPS (3, 4)**	
View 4	None	Sex, Recurrent, Response,	None
		**BDI_t1_**(1–7, **8**, 9–10, **11–15**, 16, **18–20**, 21),	
		BDI_t2_(5, 6, 8, 11, 17, 21),	
		HRSD_t1_(1–6, 8, 12, 14, 18), HRSD_t2_(4),	
		SNPS7, **MINI (1, 3)**,	
View 5	None	None	None
View 6	age, JART, age of first depression,	drug	None
	Days of current episode, HRSD17,		
	HRSD21, GAF_6w, SHAPS_6w,		
	PANAS_P_6w, BAS_6w, O, A, C,		
	Rep, BDNF, Cortisol		
View 7	None	None	None
View 8	None	**BDI_t1_(17)**, **HRSD_t2_(15)**	None
View 9	None	None	None
View 10	**BDI**, **BDI_6w**, **BDI_6m**,	HRSD_t2_(9, 19, 20),	None
	**PHQ-9**, **PHQ-9_6w**, PHQ-9_6m,	**MINI** (2, 4–11, **12**, 13–16)	
	HRSD17_6w, **HRSD21_6w**,		
	**GAF**, **SHAPS**, **PANAS_P**,		
	**PANAS_N**, **STAI**, STAI_6w,		
	**CATS:(total, N, P, E)**,		
	**LES:(total, P, N)**, BIS,		
	BAS, N, E		
View 11	None	None	None
View 12	None	Melancholic, HRSD_t1_(15),	**Episode**,
		**SNPS** (1, **2**, 6)	**RecNum**
View 13	None	None	None
View 14	None	None	None
View 15	None	None	None

Features in bold denote significant features that discriminate among subject clusters, which is evaluated by means of Analysis of Variance (ANOVA) for numerical features while for categorical and integer features by means of *χ*^2^-test for crosstable relationship. We set the level of significance to 0.05 with Bonferroni correction within each type of features. To avoid clustering, numerical features related to gene expressions are omitted. For simplicity, multiple features such as A1, A2, A3, and A5 are abbreviated as A (1–3, 5).

Next, we analyzed subject clusters. Our intent was to find meaningful subtypes of depression. Accordingly, we focused on relevance of cluster memberships to depression and separability between subject clusters. First, we evaluated relevance of subject cluster solutions for depression (Fig. 3b). In terms of the concordance between cluster memberships, the label of control/depression, and the proportion of depression related features for selected features, view 10 is the most useful for control/depression labels. Here we defined depression related features as follows: for numerical features BAS, BDI, BIS, GAF, PHQ9, HRSD, JART, PANASP, PANASN, SHAPS, STAI, N, E, O, A, and C; for categorical features drug, Melancholic, Recurrent, Response, Remission, BDI items, HRSD items, SNPs, and MINIs. In short, these features represent the current status of the psychiatric condition of a subject. In Supplementary Table S2, these features are shown with asterisks *. We did not include FC features for this definition.

Second, we evaluated separability of subject clusters of depressive subjects in FC feature clusters by means of Cohen’s *d* (Fig. 3c)^37^, which is commonly used to measure effect size of differences of two distributions. Here, we follow the criterion of effect size^37^: 0.2 small; 0.5 medium; 0.8 large. Using this criterion, subject clusters of depressive subjects in view 10 were well separated, while those in views 4, 6, 7, 8, 12, 13 and 14 were modestly so, and the remainder of the views were slightly separated. These results suggest that view 10 is specific in two regards: relevance for control/depression and large separability of depressive clusters in terms of FC.

**Figure 4:**
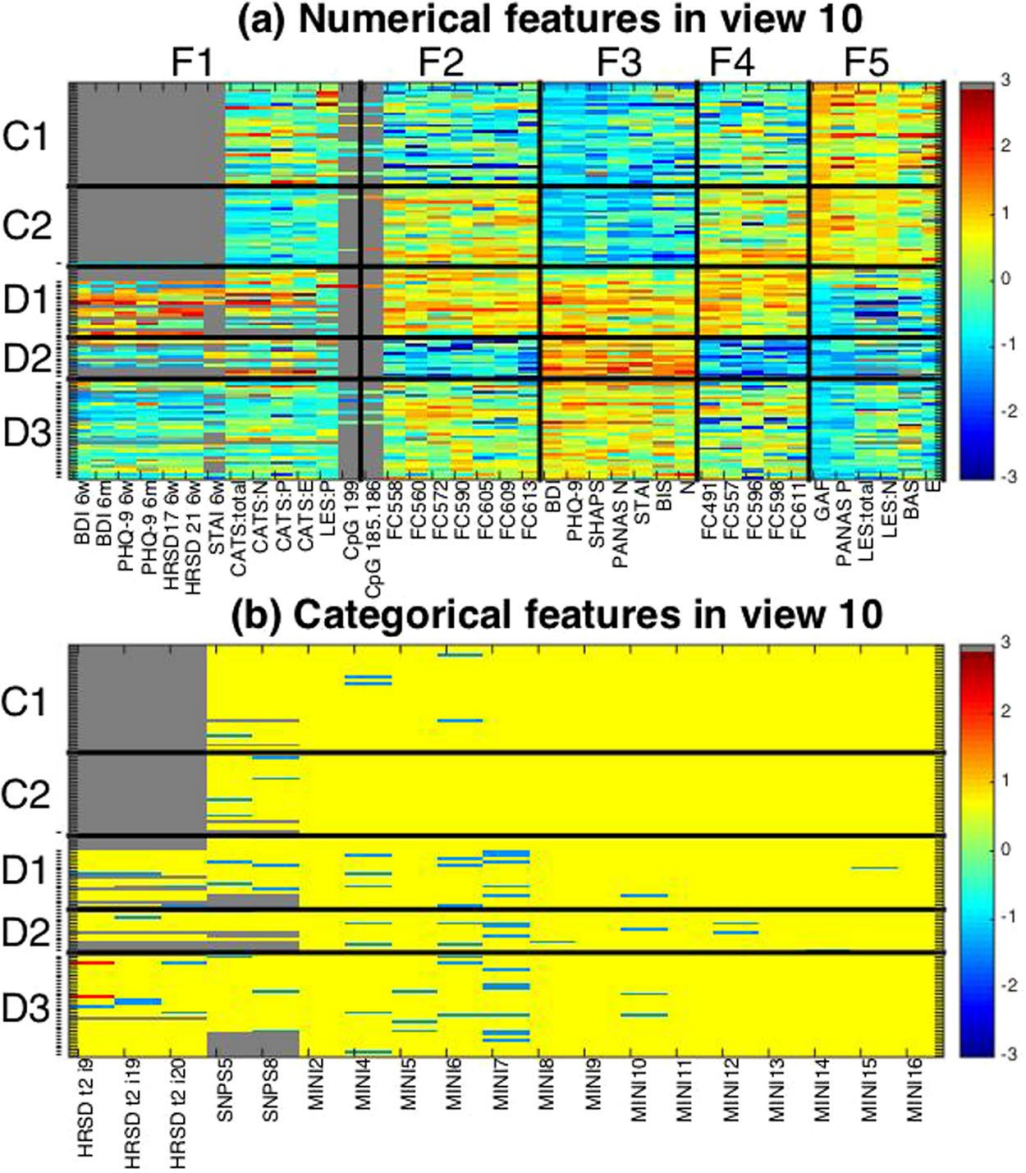
Visualization of view 10. For numerical features in Panel (a) and categorical features in Panel (b). The horizontal axis denotes features while the vertical axis denotes subjects. Depressive subjects are indicated by hyphens. Both subjects and features are sorted in the order of the subject- and feature-cluster indices. Subject clusters are named C1, C2, D1, D2, and D3 from top to bottom, while for numerical feature clusters, F1, F2, F3, F4, F5 from left to right. Subjects within a subject cluster are sorted in the order of control and depressive subjects. The color in a cell denotes the corresponding value of the data matrix indicated in the color bar where the missing entries are in gray.

**Figure 5:**
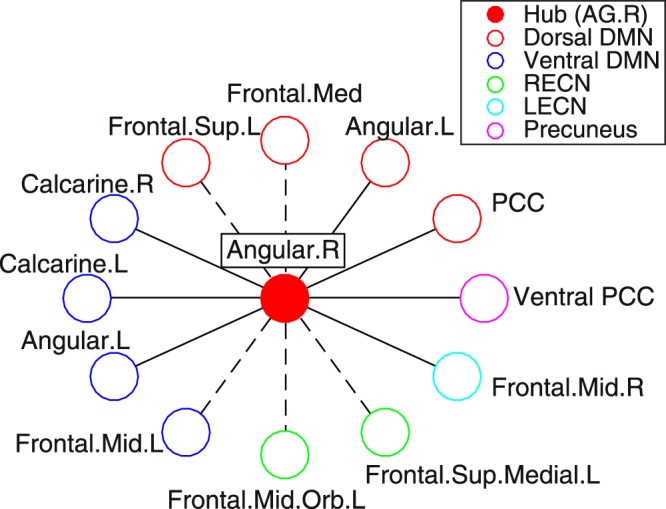
Structure of functional connectivity in feature clusters F2 (in bold line) and F4 (in dashed line). Color corresponds to intrinsic connectivity networks. Relevant brain areas for network nodes are based on Automated Anatomical Labeling (AAL). DMN denotes default mode network; RECN right executive control network; LECN left executive control network.

Further, we analyzed view 10 more in detail. This view consists of five subject clusters, which match the label of control/depression well: Two clusters for control subjects (subject clusters C1, C2) and three clusters for depressive subjects (subject clusters D1, D2, D3) (Fig. 4a). View 10 contained several non-FC features that discriminate well among subject clusters (features in bold in Table 2). Further, the view had a high proportion of depression-related features (60%), including 39 numerical features and 19 categorical features, but none of the integer features. Since the categorical features do not clearly distinguish between subject clusters (Fig. 4b), we focus only on numerical features in this view for further analysis. Numerical features are clustered into five feature clusters F1-5. We characterize each feature cluster based on characteristics of their member features (Supplementary Table S4). Since this view is closely related to the label of control/depression, it is consistent that the view has feature clusters (namely, F3 and F5) that are related to the initial status of depression. However, it is noteworthy that the view also contains a feature cluster that is related to the after-treatment status of depression (namely, F1). Furthermore, features related to CATS (Child Abuse Trauma Scale) are included in the same feature cluster F1. These results suggest that the subject clusters D1, D2 and D3 for depressive subjects may be related to after-treatment status of depression, which might be further related to stress experiences during childhood. Lastly, feature clusters F2 and F4 are related to specific functional connectivity in fMRI image data, which suggests a possible association between the subject clusters and neural substrates (Supplementary Fig. S1). In this view, the majority of these functional connections are, on average, higher for depressed patients than for controls, while the remainder of views, except for view 11, has more connectivity that is higher for controls than for depressed patients. Further, this connectivity network in view 10 forms the topology of a star with the central hub, which is also observed in other views. The relevant brain areas for the functional connectivity of view 10 are Dorsal DMN.02 (hub), Dorsal DMN.04, Dorsal DMN.06, Ventral DMN.01, Ventral DMN.05, Ventral DMN.09, LECN.01, Precuneus.01, Dorsal DMN.01, Dorsal DMN.03, Ventral DMN.07, RECN.02, and RECN.04, where DMN denotes Default Mode Network; RECN Right Executive Control network; LECN Left Executive Control network (Supplementary Fig. S2). Based on Automated Anatomical Labeling (AAL), the hub of this network is identified as the right angular gyrus (AG) while the remainder of brain areas are ACC (Anterior Cingulate Cortex). R.L, Angular.L, Calcarine.R.L, Occip.Mid.L, Frontal.Med.Orb.R.L., Frontal.Mid.R.L, Frontal.Mid.Orb.L, Frontal.Sup.R.L, Frontal.Sup.Medial.R.L, PCC (Posterior Cingulate Cortex). R.L, Precuneus.L, and Ventral PCC.R.L (Supplementary Table S5). The star topology structure of this network is visualized in Fig. 5, which shows the key role of AG in relevant default mode networks.

**Figure 6:**
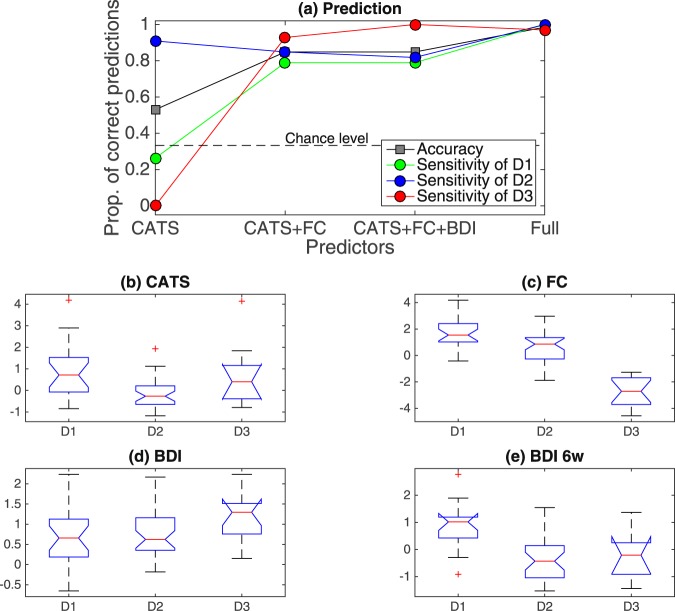
Characterization of D1, D2 and D3. Panel (a): Results of prediction of subject cluster memberships via the leave-one-out cross validation. The y-axis denotes the proportion of correct predictions of subject cluster membership: true cluster memberships are those of view 10, while predicted cluster memberships are those yielded based on finite mixture models via a leave-one-out cross validation. We carried out the leave-one-out cross validation as follows. First, we remove a particular depression subject from the data. Second, using the reduced data, we re-estimate parameters in Gaussian mixture models for specific predictors. For this parameter estimation, we use subject cluster memberships on D1, D2 and D3. Third, based on the obtained Gaussian mixture model, we estimate a cluster membership of the removed subject, which enables us to evaluate whether the model can correctly predict the cluster membership of the removed subject. Lastly, we repeat these steps for all depressive subjects, evaluating a proportion of correct predictions on cluster memberships of the depression subjects. For predictors (i.e., selected features for mixture models) in the x-axis, we started with CATS, subsequently adding functional connectivity in feature clusters F2 and F4, BDI and the remainder of features in view 10. Panels (b)–(e): Boxplots for each subject cluster in view 10. Panel (b) for CATS scores; Panel (c) for the first principal scores for FC features in feature cluster F2; Panel (d) for BDI scores; Panel (e) for after-six-week BDI scores.

In the cluster analysis of view 10, it is important to note that view 10 contains after-six-week scores such as BDI. Since view 10 also includes other features that are available before onset of treatment, this raises the possibility of prediction of treatment outcome prior to treatment. In this regard, we explore several important implications drawn from the results of cluster analysis. First, subject clusters can be represented by a small number of relevant features. Since in our clustering method each feature cluster consists of similar (i.e., highly correlated) features, a feature cluster can be represented by a reduced number of these features. It turns out that a subject cluster solution in a view can be represented by a small number of features associated with each feature cluster. View 10 (Fig. 4a) is represented by CATS scores (associated to feature cluster F1), the first principal scores of angular-gyrus related FC (associated to F2; we simply refer to it ‘AG related FC score’), and BDI (associated to F3). These features can indeed explain the resultant subject cluster membership properly (Fig. 6a). Here, we do not consider features in F4 and F5, because these features are strongly correlated with those in F2 and F3, respectively. Based on distributions of these scores in each subject cluster (Fig. 6b–e), we can characterize subject clusters as follows: subject cluster D1 by high CATS, high FC, low BDI and high BDI6w; subject cluster D2 by low CATS, moderate FC, low BDI and low BDI6w; subject cluster D3 by high CATS, low FC, high BDI and low BDI6w (Supplementary Table S6), where we also include the after-six-week BDI (BDI6w) score associated with F1. This characterization provides a basis for predicting remission of SSRI treatment that is measured by the after-six-week BDI score, because for subject cluster D1, the after-six-week BDI score is high; hence, a subject in D1 is unlikely to remit while a subject in D2 or D3 is likely to remit.

Finally, we investigate the remainder of views. We have identified non-FC features that discriminate between subject clusters based on a statistical test for individual features (features in bold in Table 2). From these results, significant correspondences between non-FC features and brain areas are found in view 4, view 8, and view 12 (Supplementary Table S7). Note that significant correspondence is also observed in view 10). These correspondences help us to interpret these views. First, view 4 includes depression items related to BDI, MINI1, and MINI3, while it also includes FC-features in which the three dominant brain areas are Language.02 (Temporal.Mid.L, in AAL), Ventral DMN.04 (Occipital.Mid.L), and Language.05 (Frontal.Inf.Orb.R) with 25, 22, and 21 connectivity, respectively. Second, view 8 includes features BDI_t1_17 and HRDS_t2_15. These items in clinical questionnaires are related to fatigue and hypochondriasis, respectively. The dominant brain area is Dorsal DMN.02 (Angular gyrus.R). Third, view 12 includes Episode and RecNum, which are related to the extent of repetitions of MDD. The dominant brain areas are Language.04 (Temporal.Mid.L), Dorsal DMN.01 (Cingulum.Ant, Frontal.Sup.Medial), and RECN.04 (Frontal.Sup.Medial).

We have so far analyzed subject cluster solutions one by one. One may wonder if it is possible to integrate all these cluster solutions, yielding a single cluster solution, which may exhibit distinct demographic, clinical and end-phenotypic characteristics. One possible way of such analysis is to carry out hierarchical clustering, using Hamming distance defined as the percentage of views in which subject cluster memberships differ (Supplementary Fig. S3). However, in the present study, we were not able to characterize the yielded clusters in a meaningful manner. Possibly, by integrating cluster solutions, useful information on cluster solutions might be lost.

## Discussion

**Figure 7:**
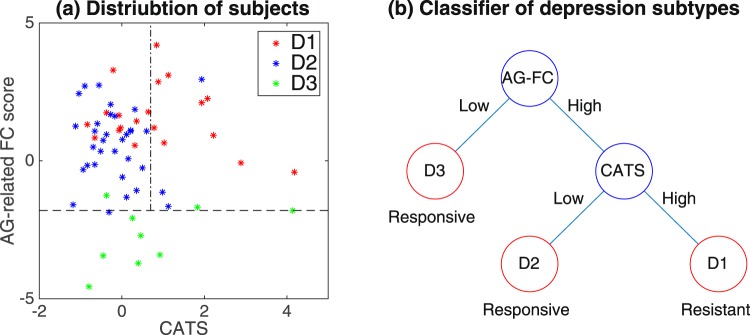
Classification of D1, D2 and D3. Panel (a): Distribution of subjects in D1, D2, and D3 for AG-related FC and CATS scores. The dashed line denotes a possible threshold of AG-related FC score to discriminate between subjects in D3 and other subjects, while the dot-dashed line denotes a possible threshold of CATS score to discriminate between subjects in D1 and D2, provided that subjects in D3 have already been sorted out. Panel (b): Classifier of subjects based on AG-related FC (AG-FC) score and CATS score. Blue circles denote classifier’s relevant features, while red ones denote resultant subject clusters. ‘Responsive’ or ‘Resistant’ signs denote whether SSRI treatment may work or not based on after-six-week BDI scores. This classifier is established based on Panel (a).

The results of the cluster analysis suggest three subtypes of depressive subjects, D1, D2, and D3 in view 10. The number of subject clusters in question is three; hence, two binary features may be sufficient for classification. In the scatter plot of subjects in D1, D2, and D3 (Fig. 7a), AG-related FC scores discriminate between subjects in D3 and other subjects. On the other hand, CATS scores do not discriminate a single class by itself, but they discriminate between subjects in D1 and D2, once subjects in D3 are sorted out. This observation motivated us to consider a classifier that consists of the following steps. First, we classify subjects into either D3 or non-D3 based on AG-related FC scores. A subject with low scores in AG-related FC is classified into D3, otherwise into non-D3. Subsequently, the non-D3 subjects are classified into either D1 or D2 based on CATS scores: A subject with low scores in CATS is classified into D2, otherwise into D1. This procedure of classification is summarized in Fig. 7b. Since these subject clusters correspond to degrees of remission of SSRI treatment as well, this classifier leads to predictions of whether SSRI treatment may be effective, prior to the onset of treatment. We can interpret this classification as follows. For subjects in D2 and D3, SSRI treatment may be appropriate (low after-six-week BDI scores, Supplementary Table S6), while for those in D1 SSRI treatment may not be a good option (high after-six-week BDI scores). Note that we do not take initial BDI scores into account in this classifier. Interestingly, the initial BDI score is not a good indicator for predictions of after-six-week BDI scores in this classification (Fig. 6d,e). For instance, for subjects in D3, the initial BDI scores are high, while after-six-week BDI scores are low, suggesting that despite having severe depression, they are likely to remit. It is notable that such a non-trivial case can be captured in our classifier based on AG-related FC score and CATS score.

Next, we interpreted implications of this classifier. For the relationship between CATS and remission, our finding is consistent with the meta analysis of previous studies^38^, which clearly suggests that experiences of child abuse trauma have a negative impact on treatment of depression. The contribution of our study in this regard is that we were able to identify among a huge number of possible associations this specific association in an unsupervised manner without prior knowledge of feature selection. As for the medical causal relationship, recent studies suggest that treatment-resistant depression may be linked to release of pre-inflammatory cytokines, which can be caused by childhood adversity^39,40^. However, in our research framework, biomarkers of inflammation were not included, which prevents us from confirming this point. Concerning the key role of the angular gyrus (AG) in predicting remission, our finding is consistent with the results of a *t*-test on differences between TRD and non-TRD^18,19^. Unfortunately, these two previous studies are contradictory because the former study suggests that a higher FC is associated with TRD, while the latter study found that a lower FC is associated with TRD. Our result supports the former study. Moreover, recent studies have revealed that AG is related to several functions, such as semantic processing, default mode network, number processing, attention and spatial cognition, and memory retrieval^41^. Such multiple functions of AG are consistent with an fMRI study^42^, which suggests that AG is one of the major connecting hubs, together with the occipital and ventral-medial parietal. Nonetheless, these results of previous studies do not adequately explain the possible association between AG and remission of depression implied in our study. Further research is required for clarification of this point, which may provide useful insights into possible treatment of depressive patients by means of neurofeedback.

Lastly, we provide possible interpretations for views 4, 8, and 12 in which significant correspondences between non-FC features and brain areas have been found. We base our interpretations on previous studies of relevant brain areas. First, for view 4, the relevance of the identified brains areas, temporal, occipital, and frontal orbital cortex, to depression is suggested in terms of cell communication^43^, concentration of *γ*-Aminobutyric acid (GABA)^44^, and volume^45^, respectively. Also taking into account that this view includes depression-related features that discriminate subject clusters, we interpret this to mean that view 4 represents subject clusters of different levels of depressive symptoms (note that view 4 is related to depression in terms of subject clusters in Fig. 3b). Second, we have found that view 8 is characterized by non-FC features related to fatigue and FC features of angular gyrus. In previous studies, it is reported that the angular gyrus modulates *α* rhythm^46^, which is related to fatigue^47^. Hence, we interpret that to mean that view 8 represents subject clusters related to fatigue. Third, view 12 includes repetitions of depression as non-FC features while temporal, anterior cingulum cortex, and frontal cortex are viewed as FC features. It is reported that neuroplastic change occurs during depressive episodes in dorsomedial prefrontal cortex, dorsolateral prefrontal cortex, and anterior cingulum^48^, which matches our results. Hence, we believe that view 12 represents subject clusters related to the duration of episodes of depression.

Finally, we discuss limitations of the present study. First, we did not include the cerebellum because the image on this brain region was not reliably obtained. However, several resting state fMRI studies suggest that the cerebellum plays a key role in depression^49–51^. Second, from a statistical point of view, the sample size in our study is rather small. To generalize the results of the present study, further research is required with much larger sample size, which might be possible to be obtained by combining samples from different studies. Lastly, we should mention that our data had a significant difference in age between depressive and control subjects (Table 1) unlike usual case-control studies on psychiatric disorders. In fact, the bias could correlate with a risk and/or a severity of depression to some degree, as the feature ‘age’ was assigned to the same view as several clinical scores of depression symptoms in our clustering result (view 6 in Table 2). However, this view was independent of that containing AG-related FC score and CATS score (view 10 in Table 2), suggesting that the age bias has little influence on our main results.

## Electronic supplementary material

Supplementary Information

## Data Availability

Due to potentially identifying information, the data for this study are ethically restricted by the Ethical Committee for Epidemiology of Hiroshima University, Japan. Interested, qualified researchers may request the data by contacting Dr. Shoji Karatsu (kasumi-kenkyu@office.hiroshimau.ac.jp).
