# Supplementary material for: Identification of depression subtypes and relevant brain regions using a data-driven approach
Source: Sci Rep. 2018 Sep 20;8:14082. doi: 10.1038/s41598-018-32521-z (PMC6148252; doi:10.1038/s41598-018-32521-z)
Supplement: Supplementary file 1 — Supplementary Information [file 41598_2018_32521_MOESM1_ESM.pdf]

Supplementary Information for "Identification of depression subtypes and relevant brain regions using a data-driven approach"

Tomoki Tokuda[1,*], Junichiro Yoshimoto[3, 1], Yu Shimizu[1], Go Okada[2], Masahiro Takamura[2], Yasumasa Okamoto[2], Shigeto Yamawaki[2], and Kenji Doya[1]

[*]tomoki.tokuda@oist.jp
[1]Okinawa Institute of Science and Technology Graduate University, 1919-1 Tancha, Okinawa, 904-0495, JAPAN
[2]Department of Psychiatry and Neurosciences, Hiroshima University, 1-2-3 Kasumi, Minami-ku, Hiroshima, 734-8851, JAPAN
[3]Graduate School of Information Science, Nara Institute of Science and Technology, 8916-5 Takayama, Ikoma, Nara 630-0192, JAPAN

Multiple co-clustering method

This method is based on nonparametric Bayesian mixture models in which features are automatically partitioned into views for each clustering solution of subtypes. This feature partition works as feature selection for a particular clustering solution, which screens out irrelevant features. For each view, a co-clustering structure is introduced, which enables to fit to high-dimensional data. This method uses a hierarchical Dirichlet process to generate feature memberships: first allocate a feature to a view; second allocate to a feature cluster in that view. Moreover, mixing of several types of features is allowed, such as mixtures of Gaussian, categorical, and Poisson distributions, which are pre-specified by the user. In the following sub-sections, we formulate the model assumed in this method, using the notation in Supplementary Table S1. For more details of this method, please refer to the original paper[1].

Model

We assume that a data matrix X consists of M distribution families that are known in advance. We decompose $X = \{X^{(1)}, \ldots, X^{(m)}, \ldots, X^{(M)}\}$ with data size $n \times d^{(m)}$ for $X^{(m)}$, where m is an indicator for a distribution family ($m = 1, \ldots, M$). Further, we denote the number of views as V (common to all distribution families), the number of feature clusters $G_v^{(m)}$ for view v and distribution family m, and the number of object clusters K_v for view v (common to all distribution families). Moreover, for simplicity of notation, we use $G^{(m)} = \max_v G_v^{(m)}$ and $K = \max_v K_v$ to denote the number of features and the number of clusters, allowing for empty clusters.

With this notation, for i.i.d. $d^{(m)}$-dimensional random vectors $X_1^{(m)}, \ldots, X_n^{(m)}$ for distribution family m, we consider a $d^{(m)} \times V \times G^{(m)}$ feature-partition tensor (3rd-order) $Y^{(m)}$ in which $Y_{j,v,g}^{(m)} = 1$ if feature j of distribution family m belongs to feature cluster g in view v (0 otherwise). Combining this for different distribution families, we let $Y = \{Y^{(m)}\}_m$. Similarly, we consider a $n \times V \times K$ object-partition (3rd-order) tensor Z in which $Z_{i,v,k} = 1$ if object i belongs to object cluster k in view v. Note that feature j belongs to one of the views (i.e., $\sum_{v,g} Y_{j,v,g}^{(m)} = 1$) while object i belongs to each view (i.e., $\sum_k Z_{i,v,k}^{(m)} = 1$). Further, Z is common to all distribution families, which implies that our model estimates subject cluster solutions using information on all distribution families.

For a prior generative model of Y, we consider a hierarchical structure of views and feature clusters: views are first generated, followed by generation of feature clusters. Thus, features are partitioned in terms of pairs of view and feature cluster memberships, which implies that the allocation of feature is jointly determined by its view and feature cluster. On the other hand, objects are partitioned into object clusters in each view, hence, we consider just a single structure of object clusters for Z. We assume that these generative models are all based on a stick-breaking process as follows.

Generative model for feature clusters Y

We let $Y_{j\cdot\cdot}^{(m)}$ denote a view/feature cluster membership vector for feature j of distribution family m, which is generated by a hierarchical stick-breaking process:

$$
\begin{aligned}
w_v &\sim \mathrm{Beta}(\cdot|1, \alpha_1), \; v = 1, 2, \ldots \\
\pi_v &= w_v \prod_{t=1}^{v-1} (1 - w_t), \\
{w'}_{g,v}^{(m)} &\sim \mathrm{Beta}(\cdot|1, \alpha_2), \; g = 1, 2, \ldots, m = 1, \ldots, M \\
{\pi'}_{g,v}^{(m)} &= {w'}_{g,v}^{(m)} \prod_{t=1}^{g-1} (1 - {w'}_{t,v}^{(m)}), \\
\tau_{g,v}^{(m)} &= \pi_v {\pi'}_{g,v}^{(m)} \\
Y_{j\cdot\cdot}^{(m)} &\sim \mathrm{Mul}(\cdot|\tau^{(m)}),
\end{aligned}
$$

where $\tau^{(m)}$ denotes a $1 \times GV$ vector $(\tau_{1,1}^{(m)}, \ldots, \tau_{G,V}^{(m)})^T$ (the superscript T denotes matrix transposition); $\mathrm{Mul}(\cdot|\pi)$ is a multinomial distribution of one sample size with probability parameter π; $\mathrm{Beta}(\cdot|a, b)$ is a Beta distribution with prior sample size (a, b); $Y_{j\cdot\cdot}^{(m)}$ is a $1 \times GV$ vector $(Y_{j,1,1}^{(m)}, \ldots, Y_{j,V,G}^{(m)})^T$. Note that we truncate the number of views with sufficient large V and the number of feature clusters with G. When $Y_{j,v,g}^{(m)} = 1$, feature j belongs to feature cluster g at view v. By default, we set the concentration parameters α_1 and α_2 to one.

Generative model for object clusters Z

A subject cluster membership vector of object i in view v, denoted as $Z_{i,v\cdot}$, is generated by

$$
\begin{aligned}
u_{k,v} &\sim \mathrm{Beta}(\cdot|1,\beta),\ v=1,2,\ldots,\ k=1,2,\ldots \\
\eta_{k,v} &= u_{k,v}\prod_{t=1}^{k-1}(1-u_{t,v}), \\
Z_{i,v\cdot} &\sim \mathrm{Mul}(\cdot|\eta_v),
\end{aligned}
$$

where $Z_{i,v\cdot}$ is a $1\times K$ (we take K sufficiently large) vector given by $Z_{i,v\cdot}=(Z_{i,v,1},\ldots,Z_{i,v,K})^T$. We set the concentration parameter β to one.

Likelihood and prior distribution

We assume that each instance $X_{i,j}^{(m)}$ independently follows a certain distribution, conditional on Y and Z. We denote $\theta_{v,g,k}^{(m)}$ as parameters of distribution family m in the cluster block of view v, feature cluster g and object cluster k. Further denoting $\Theta=\{\theta_{v,g,k}^{(m)}\}_{v,g,k,m}$, the logarithm of likelihood of X is given by

$$
\log p(X|Y,Z,\Theta)=\sum_{m,v,g,k,j,i}\mathbb{I}(Y_{j,v,g}^{(m)}=1)\mathbb{I}(Z_{i,v,k}=1)\log p(X_{i,j}^{(m)}|\theta_{v,g,k}^{(m)}),
$$

where $\mathbb{I}(x)$ is an indicator function, i.e, returning 1 if x is true, and 0 otherwise. Note that the likelihood is not directly associated with $w=\{w_v\}_v$, $w'=\{w'^{(m)}_{g,v}\}_{g,v}$ and $u=\{u_{k,v}\}_{k,v}$. The joint prior distribution of unknown variables $\phi=\{Y,Z,w,w',u,\Theta\}$ (i.e., class membership variables and model parameters) is given by

$$
p(w)p(w')p(Y|w,w')p(u)p(Z|u)p(\Theta).
$$

Observation models

For observation models, we consider Gaussian, categorical, and Poisson distributions. For each cluster block, we fit a univariate distribution of these families with the assumption that features within the cluster block are independent. We assume conjugate priors for the parameters of these distribution families as follows.

Gaussian distribution

We denote univariate Gaussian density function as $\mathrm{Gauss}(\cdot|\mu,\sigma^2)$ where μ and σ^2 are mean and variances. We assume conjugate priors for μ and σ^2 in each cluster block:

$$
\begin{aligned}
s_{v,g,k} &\sim \mathrm{Ga}(\cdot|\gamma_0/2,\gamma_0\sigma_0^2/2) \\
\mu_{v,g,k} &\sim \mathrm{Gauss}(\cdot|\mu_0,(\lambda_0 s_{v,g,k})^{-1}),
\end{aligned}
$$

where $\mathrm{Ga}(\cdot|a,b)$ denotes Gamma distribution with shape and rate parameters (a,b). In the present paper, we set $\sigma_0^2=1/100$, $\gamma_0=1/100$, and $\lambda_0=1/100$ so that the prior distributions are nearly non-informative.

Categorical distribution

For a categorical feature x ($x\in\{c_1,\ldots,c_H\}$), we denote categorical distribution as $\mathrm{Cat}(\cdot|p)$ where H is the number of categories, and $p=(p_1,\ldots,p_H)$ are probabilities for each category with $\sum_{h=1}^H p_h=1$. We assume the conjugate prior for $(p_1,\ldots,p_H)$,

$$
(p_1,\ldots,p_H) \sim \mathrm{Dirichlet}(\cdot|\rho_0),
$$

where $\mathrm{Dirichlet}(\cdot|\rho_0)$ denotes a Dirichlet distribution with prior sample size ρ_0. We set ρ_0 to $(1,\ldots,1)$.

Poisson distribution

We denote Poisson distribution as $\mathrm{Poisson}(\cdot|\lambda)$ where λ is a rate parameter. The conjugate prior for λ is given by

$$
\lambda_{v,g,k} \sim \mathrm{Ga}(\cdot|\alpha_0,\beta_0),
$$

where we set α_0 and β_0 to one.

Sensitivity Analysis

We examined sensitivity of the clustering results to the setting of hyperparameters. For stick-breaking process, we focussed on all three hyperparameters α_1, α_2 and β while for hyperparameters in probabilistic probabilities, we focussed only on $\gamma_0, \sigma_0^2, \lambda_0, \mu_0$ in Gaussian priors, because the numerical features outnumber the remainder of the features, hence, most influential for the clustering results. In our context, however, sensitivity analysis would be computationally demanding: For a single perturbation of hyperparameters, it would take 160 hrs to obtain optimal clustering results. To reduce computational costs, we focussed only on the initial configuration that gave us the optimal clustering solution for the original setting of hyperparameters (it takes only 0.16 hrs). Further, we separately analyzed hyperparameters in stick-breaking process and Gaussian distributions. We carried out sensitive analysis by means of grid search[2]. We set the ranges of $\alpha_1, \alpha_2,$and β to [0.5, 1, 2] while the ranges of $\gamma_0, \sigma_0^2,$and λ_0 to [1/1000, 1/100, 1/100], and μ to [-0.1, 0, 0.1], respectively. Note that in all cases, the hyperparameters are perturbed both in negative and positive directions. This setting requires $3^3 + 3^4 = 108$ runs of application of the multiple co-clustering method. To evaluate concordance between obtained results, we focussed on a subject cluster solution in view 10, which plays the key role in the present paper. We have the following results. For hyperparameters of stick-breaking process, in all cases there is a good accordance of subject cluster solutions: Adjuster Rand Index (ARI)[3] takes more than 0.8 between the original hyperprameters and the perturbed hyperparameters (ARI takes one for perfect accordance between two cluster solutions while zero for random accordance); for Gaussian hyperparameters, in 85 % of cases, ARI takes more than 0.8. There results suggest that the clustering results are not sensitive to a small perturbation of our setting of hyperparameters.

References

1. Tokuda, T. *et al.* Multiple co-clustering based on nonparametric mixture models with heterogeneous marginal distributions. *PLoS ONE* **12**, e0186566; 10.1371/journal.pone.0186566 (2017).

2. Roos, M., Held, L. *et al.* Sensitivity analysis in Bayesian generalized linear mixed models for binary data. *Bayesian Analysis* **6**, 259–278 (2011).

3. Hubert, L. & Arabie, P. Comparing partitions. *J. Classif.* **2**, 193–218 (1985).

Table S1. Notation for multiple clustering model

Domain	Notation	Description
Data	n	Sample size
	m	m^{th} distribution family ($m = 1, \ldots, M$)
	M	Total number of distribution families
	$d^{(m)}$	Number of features for distribution family m
	$X^{(m)}$	Data matrix for distribution family m of size $n \times d^{(m)}$
	$X_i^{(m)}$	i^{th} sample for distribution family m of size $1 \times d^{(m)}$
	X	All data matrix of size $n \times \sum_{m=1}^{M} d^{(m)}$
Cluster Membership	V	Number of views
	$G_v^{(m)}$	Number of feature clusters for distribution family m in view v
	K_v	Number of object clusters in view v
	$G^{(m)}$	$\max_v G_v^{(m)}$
	K	$\max_v K_v$
	$Y^{(m)}$	Feature-partition indicators of size $d^{(m)} \times V \times G^{(m)}$
	$Y_{j..}^{(m)}$	Feature-partition indicators for feature j of distribution family m of size $V \times G^{(m)}$
	$Y_{j,v,g}^{(m)}$	Element of $Y^{(m)}$: 1 if feature j of distribution family m belongs to cluster g in view v, or 0 otherwise
	Z	Object-partition indicators of size $n \times V \times K$
	$Z_{i,v.}$	Object-partition indicators for object i in view v of size $1 \times K$
	$Z_{i,v,k}$	Element of Z: 1 if object i belongs to object cluster k in view v, or 0 otherwise
Dirichlet Process	w_v	Probability of stick-breaking for view v
	α_1	Hypeparameter of a beta prior Beta(1, α_1) for w_v
	π_v	Length of unit-stick ($\sum_{v=1}^{\infty} \pi_v = 1$) for view v
	$w'^{(m)}_{g,v}$	Probability of stick-breaking for feature cluster g for distribution family m in view v
	α_2	Hypeparameter of a beta prior Beta(1, α_2) for $w'^{(m)}_{g,v}$
	$\pi'^{(m)}_{g,v}$	Length of unit-stick ($\sum_{g=1}^{\infty} \pi'^{(m)}_{g,v} = 1$) for feature cluster g of distribution family m in view v
	$\tau_{g,v}^{(m)}$	$\pi_v \pi'^{(m)}_{g,v}$: Length of unit-stick ($\sum_{g,v}^{\infty} \tau_{g,v}^{(m)} = 1$) for feature cluster g of distribution family m in view v
	$u_{k,v}$	Probability of stick-breaking for object cluster k in view v
	β	Hypeparameter of a beta prior Beta(1, β) for $u_{k,v}$
	$\eta_{k,v}$	Length of unit-stick ($\sum_{k=1}^{\infty} \eta_{k,v} = 1$) for object cluster k in view v
Probability Model	$\theta_{v,g,k}^{(m)}$	Parameter(s) of distribution family m for feature cluster g and object cluster k in view v

Table S2. List of features for clinical data (non-FC features). The features with an asterisk * are considered as depression related features in Fig.3b in the main manuscript.

**Numerical features**
age
age when first depressive symptoms show up,
The number of days elapsed for current episode,
BAS* (Behavioral Activation Scale),
BDNF (Quantity of brain-derived neurotrophic factor in blood),
BDI* (Beck Depression Inventory),
BIS* (Behavioral Inhibition Scale),
CATS (Child Abuse and Trauma Scale),
CATS:total for all items
CATS:N for items on neglect
CATS:S for items on sexual abuse
CATS:P for items on punishment
CATS:E for items on emotion
Cortisol (Quantity of cortisol in blood),
CpG_, S_, SU_, SD_ (Methylation probability),
FC1-2701 (Functional connectivity),
GAF* (Global Assessment of Functioning),
PHQ9* (Patient Health Questionnaire),
HRSD17* (17-item Hamilton Rating Scale for Depression),
HRSD21* (21-item Hamilton Rating Scale for Depression),
HRSDchange (Increment rate of HRSD17 scores defied as $(\mathrm{HRSD176w} - \mathrm{HRSD17})/\mathrm{HRSD17}$).
JART* (Adult reading test),
LES (Life Experiences Survey),
LES:total for all events
LES:P for positive events
LES:N for negative events
PANASP* (Positive Affect Schedule),
PANASN* (Negative Affect Schedule),
SHAPS* (Snaith-Hamilton Pleasure Scale),
STAI* (State-Trait Anxiety Inventory),
N*, E*, O*, A*, C* (Five factors in revised NEO Personality Inventory)
**Categorical features**
BDI*_t1_ (Items of BDI initially),
BDI*_t2_ (Items of BDI after six weeks of treatment),
drug* (states of dosing of lexapro),
HRSD*_t1_(items of HRSD initially)*,
HRSD*_t2_(items of HRSD after six weeks of treatment)*,
Melancholic* (Melancholic depression or not),
MINI* (Mini-International Neuropsychiatric Interview): the numbering corresponds to the following psychiatric symptoms.
Major depressive disorder (1),
Dysthymia (2), Suicide risk (3),
Mania (4), Panic disorder (5), Agoraphobia (6),
Social phobia (7),
Obsessive compulsive disorder (8),
PTSD (9), Alcohol dependence and abuse (10),
Drug dependence and abuse (11),
Psychotic disorder (12), Anorexia (13), Bulimia (14),
Generalized anxiety disorder (15),
Antisocial personality disorder (16),
Recurrent* (Recurrent depression or not),
Response* (whether there is response to the reatment based on HRSD17),
Remission* (whether a patient is remitted after the tremens),
Sex
SNPs 1-8: Single Nucleotide Polymorphisms that are located in the following genome sites, respectively. (in parenthesis are the relevant gene functions)*
rs1187323 (NTRK2), rs34118353 (5HT1a receptor), rs3756318 (NTRK2), rs3813929 (5HT2c receptor), rs45554739 (NTRK2), rs56384968 (SLC6A4), rs6265 (BDNF), rs6294 (5HT1a receptor)
**Integer features**
Episode (the number of past experiences of depression),
RecNum (the number of times of recurrent depression)

Table S3. Results of multiple co-clustering. The number of clusters (denoted as # clusters) for subjects, numerical features, categorical features, and integer features; the number of features (denoted as # features) for each type of features. Views are sorted in descending order of the total number of features included.

View ID	Subject	Feature					
		Numerical		Categorical		Integer	
	# clusters	# clusters	# features	# clusters	# features	# clusters	# features
View 1	9	11	1283	1	4	0	0
View 2	9	11	396	1	20	0	0
View 3	7	6	316	1	17	0	0
View 4	7	6	221	3	46	0	0
View 5	7	6	144	0	0	0	0
View 6	6	5	117	1	1	0	0
View 7	5	3	66	0	0	0	0
View 8	4	3	62	1	2	0	0
View 9	6	2	62	0	0	0	0
View 10	5	5	39	1	19	0	0
View 11	5	2	43	0	0	0	0
View 12	4	2	32	2	5	1	2
View 13	4	1	35	0	0	0	0
View 14	4	1	15	0	0	0	0
View 15	3	1	1	0	0	0	0

Table S4. Characteristics of feature clusters in view 10. We use a serial number for identification of FC features, which is clarified in Supplementary Table S5.

Feature-cluster	Member feature	Characteristic
F1	BDI_6w, BDI_6m, PHQ-9_6w, PHQ-9_6m, HRSD17_6w, HRSD 21_6w, STAI_6w, CATS:total, CATS:N, CATS:P, CATS:E, LES:P, CpG 199	After-treatment status & Child abuse trauma
F2	FC558, FC560, FC572, FC590, FC605, FC609, FC613, CpG 185.186,	Functional connectivity 1
F3	BDI, PHQ-9, SHAPS, PANASN, STAI, BIS, N	Initial status 1
F4	FC491, FC557, FC596, FC598, FC611	Functional connectivity 2
F5	GAF, PANASP, LES:total, LES:N, BAS, E	Initial status 2

Table S5. Relevant brain areas for view 10. All FC has connectivity with Dorsal.DMN.02 (Angular.R). In the third column, the remainder of network nodes are displayed.

Feature-cluster	FC ID	Network node	Number of connection	Relevant brain areas (AAL)	Brodmann Area
F2	NA	Dorsal.DMN.02	7	Angular.R	39
	558	Dorsal.DMN.04	1	PCC (Posterior Cingulate Cortex).R.L	23
	560	Dorsal.DMN.06	1	Angular.L	39
	605	Ventral.DMN.01	1	Calcarine.R	30
	609	Ventral.DMN.05	1	Calcarine.L, Precuneus.L	17, 23, 29, 30
	613	Ventral.DMN.09	1	Angular.L, Occipital.Mid.L	39
	572	LECN.01	1	Frontal.Mid.R	8
	590	Precuneus.01	1	Ventral PCC.R.L	23
F4	NA	Dorsal.DMN.02	5	Angular.R	39
	491	Dorsal.DMN.01	1	Frontal.Sup.Medial.R.L, ACC (Anterior Cingulate Cortex).R.L, Frontal.Med.Orb.R.L, Frontal.Sup.R	10
	557	Dorsal.DMN.03	1	Frontal.Sup.L	9
	611	Ventral.DMN.07	1	Frontal.Mid.L	NA
	596	RECN.02	1	Frontal.Mid.Orb.L, Frontal.Mid.L	46
	598	RECN.04	1	Frontal.Sup.Medial.L	8

Table S6. Characterization of subject-clusters with values of relevant features. 'High', 'Moderate', and 'Low' in the table denotes the level of mean values of these features in each subject cluster.

Subject-	Features			
cluster	CATS	FC	BDI	BDI6w
D1	High	High	Low	High
D2	Low	Moderate	Low	Low
D3	High	Low	High	Low

Table S7. Relevant non FC and FC features in views 4, 8, and 12. Discriminative features are those significantly discriminate subject clusters, which are denoted in bold in Table 1 in the main manuscript.

	Non-FC features		FC-features	
View ID	Discriminative Feature	Description	Dominant Brain areas	# connectivity
View 4	BDI_t1_i(8, 11-15, 18-20)	Depression	La-2, Ve-4, La-5	25, 22, 21
	MINI1	Depression		
	MINI3	Suicidal risk		
View 8	BDI_t1_17	Fatigue	Do-2, Re-6	43, 2
	HRDS_t2_15	Hypochondriasis		
View 12	SNPS2	DNA	La-4, Do-1, Re-4	6, 5, 5
	Episode	# past depression		
	RecNum	# recurrent depression		

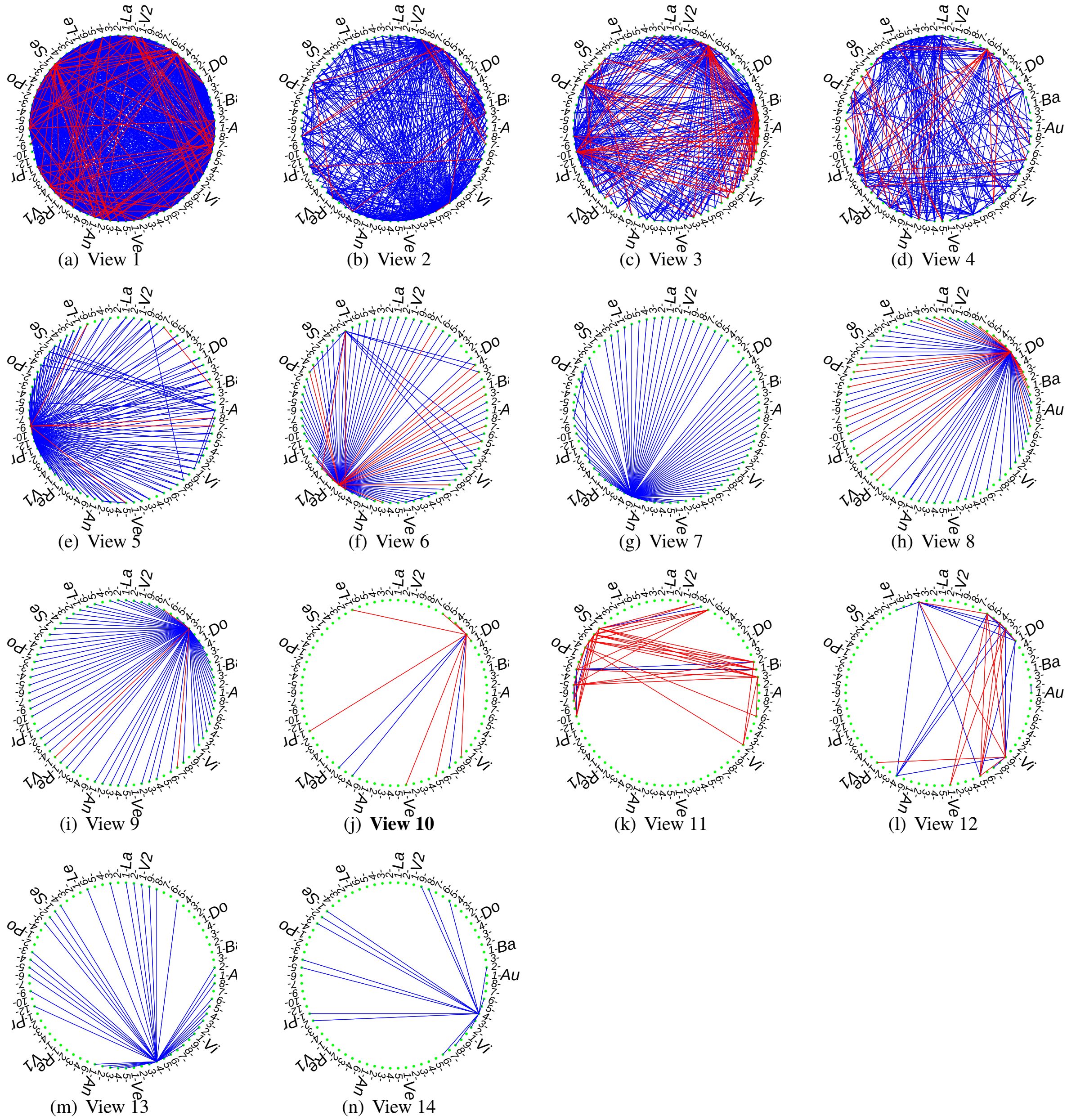

(a) View 1 (b) View 2 (c) View 3 (d) View 4 (e) View 5 (f) View 6 (g) View 7 (h) View 8 (i) View 9 (j) **View 10** (k) View 11 (l) View 12 (m) View 13 (n) View 14

Figure S1. Relevant functional connectivity for views. From Panel (a) to Panel (n), views 1-14, respectively (view 15 is omitted because it does not include FC features). Selected functional connectivity between two brain networks in each view are denoted in red if the average of depressive subjects is higher than the average of control subjects, and otherwise in blue. Acronym for brain networks in nodes: An (Anterior Salience), Au (Auditory), Ba (Basal Ganglia), Do (Dorsal Default Mode), La (Language), Le (Left Executive Control), Pr (Precuneus), Po (Posterior Salience), Re (Right Executive Control), Ve (Ventral Default Mode), Vi (Visuospatial), V1 (Primary Visual), V2 (Higher Visual), and Se (Sensorimotor). For the numbering after these acronyms in an anticlockwise way, see http://findlab.stanford.edu/functional_ROIs.html.

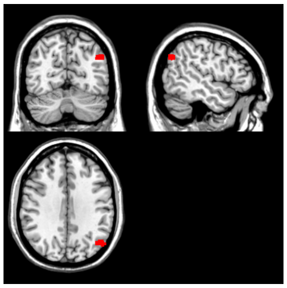

(a) ***Dorsal DMN.02***

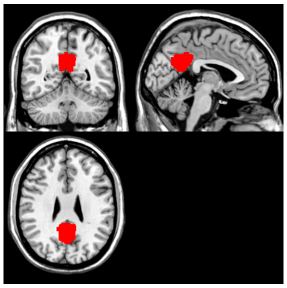

(b) **Dorsal DMN.04**

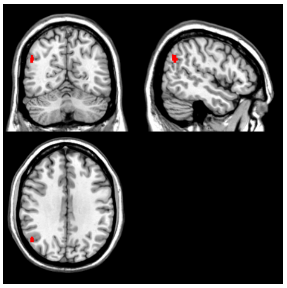

(c) **Dorsal DMN.06**

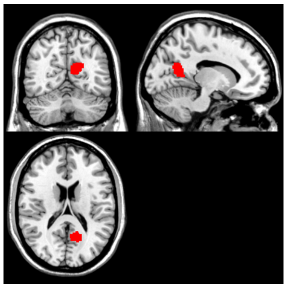

(d) **Ventral DMN.01**

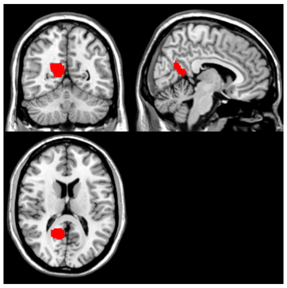

(e) **Ventral DMN.05**

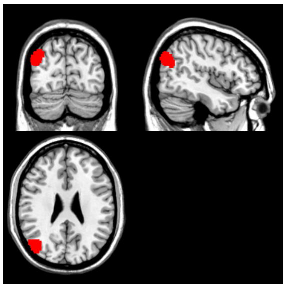

(f) **Ventral DMN.09**

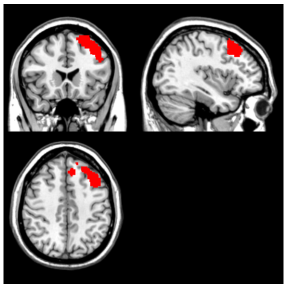

(g) **LECN.01**

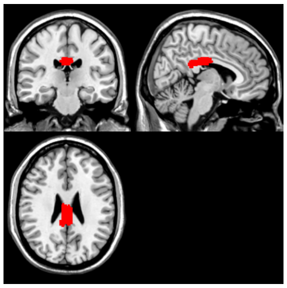

(h) **Precuneus.01**

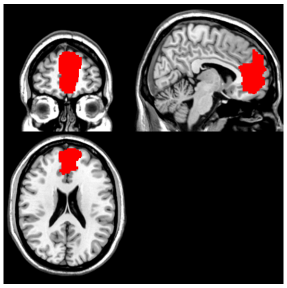

(i) *Dorsal DMN.01*

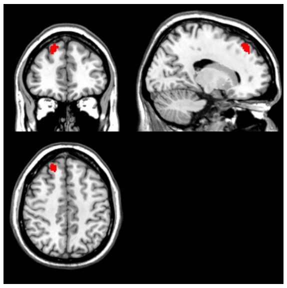

(j) *Dorsal DMN.03*

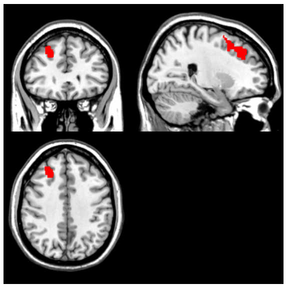

(k) *Ventral DMN.07*

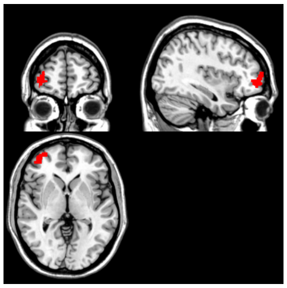

(l) *RECN.02*

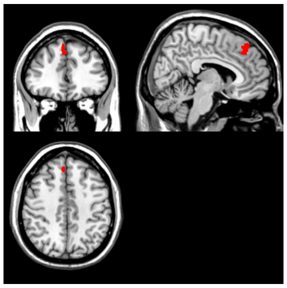

(m) *RECN.04*

Figure S2. Images of relevant brain areas for view 10. For Panel (a), the right angular gyrus, which plays the role of the hub in both functional connectivity clusters F2 and F4 (denoted in bold italic). For Panels (b)-(h), the remainder of relevant brain areas in F2 (denoted in bold); for Panels (i)-(m), the remainder of relevant brain areas in F4 (denoted in italic). These images were obtained by MRIcro (www.mricro.com) using nii files for relevant brain areas available at http://findlab.stanford.edu/functional_ROIs.html.

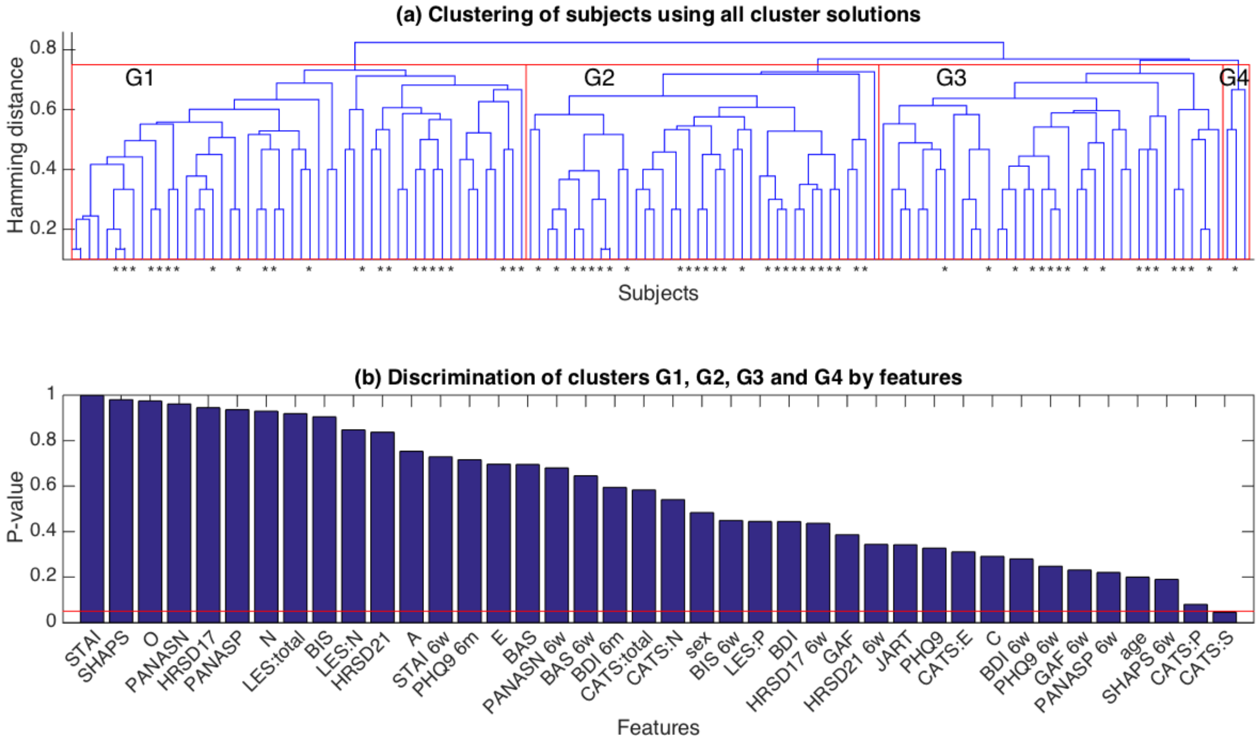

Figure S3. Integration of 15 clustering solutions of subjects. Panel (a): Results of hierarchical clustering measuring Hamming distance between two subjects, defined as the percentage of views in which the subject cluster memberships differ. The average linkage method is used for merging two clusters. Subject clusters G1, G2, G3, and G4 as enclosed in red, were identified with cutoff value 0.75 in Hamming distance. The star marked subjects denote depressive subjects. Panel (b): Results of statistical test to examine discrimination of clusters G1-G4 by features. For numerical features, we considered clinical scores and age, while sex for categorical one. We evaluated p-value of Kruskal-Wallis test (nonparametric version of ANOVA) for each numerical feature and p-value of χ^2-test for categorical one. In the panel, features are sorted in descending order of p-values. The red line denotes the threshold 0.05 for p-value without Bonferroni correction.
